# Predicted Impact of Barriers to Migration on the Serengeti Wildebeest Population

**DOI:** 10.1371/journal.pone.0016370

**Published:** 2011-01-25

**Authors:** Ricardo M. Holdo, John M. Fryxell, Anthony R. E. Sinclair, Andrew Dobson, Robert D. Holt

**Affiliations:** 1 Division of Biology, University of Missouri, Columbia, Missouri, United States of America; 2 Department of Integrative Biology, University of Guelph, Guelph, Ontario, Canada; 3 Department of Zoology, University of British Columbia, Vancouver, British Columbia, Canada; 4 Department of Ecology and Evolutionary Biology, Princeton University, Princeton, New Jersey, United States of America; 5 Department of Zoology, University of Florida, Gainesville, Florida, United States of America; University of Zurich, Switzerland

## Abstract

The Serengeti wildebeest migration is a rare and spectacular example of a once-common biological phenomenon. A proposed road project threatens to bisect the Serengeti ecosystem and its integrity. The precautionary principle dictates that we consider the possible consequences of a road completely disrupting the migration. We used an existing spatially-explicit simulation model of wildebeest movement and population dynamics to explore how placing a barrier to migration across the proposed route (thus creating two disjoint but mobile subpopulations) might affect the long-term size of the wildebeest population. Our simulation results suggest that a barrier to migration—even without causing habitat loss—could cause the wildebeest population to decline by about a third. The driver of this decline is the effect of habitat fragmentation (even without habitat loss) on the ability of wildebeest to effectively track temporal shifts in high-quality forage resources across the landscape. Given the important role of the wildebeest migration for a number of key ecological processes, these findings have potentially important ramifications for ecosystem biodiversity, structure, and function in the Serengeti.

## Introduction

The Serengeti wildebeest migration is a unique part of our biological heritage. Large-scale ungulate migrations, now rare, were once commonplace across the globe [1], [2], [3], [4]. Many migrations, such as those of the Great Plains bison, the wildebeest and springbok of southern Africa, and most recently the saiga antelope on the Russian steppes, have collapsed in historic times [1]. Landscape fragmentation and the construction of man-made barriers to movement are widely considered to have contributed to such declines [1], [2], [5]. The Serengeti is a rare example where – through brilliant foresight – a large-scale ungulate migration has been saved because of well-executed reserve design.

The Government of Tanzania has recently announced plans to construct an all-weather road bisecting the northern portion of Serengeti National Park [6]. Concerns have been raised that such a road might truncate the wildebeest migration with disastrous consequences for the carrying capacity of this species in the system, leading to direct and indirect effects impacting many other species and ecosystem processes [6]. To understand quantitatively how disrupting the migration might impact the wildebeest population, it is first necessary to characterize how mobility and the ability to track spatially- and temporally-varying resources contribute to sustain migratory ungulates in this ecosystem, compared to their sedentary counterparts. Forage quality and food intake peak at intermediate levels of grass biomass [7], [8], [9], [10], [11], and migratory ungulates are effective at finding high-quality forage patches across heterogeneous landscapes in a range of ecosystems [5], [10], [11], including the Serengeti [9], [12], [13], [14]. This ability to track transient areas of high productivity across the landscape translates into a demographic advantage for migratory animals over sedentary ones [11], [15]. Landscape fragmentation, by disrupting movement patterns and lowering the efficiency of resource use over the annual cycle, can lead to reduced population growth and a lower carrying capacity for migratory ungulates in landscapes with high functional heterogeneity [5], [13], [15], [16], [17].

Previous models have explored the importance of resource heterogeneity in the context of migration in the Serengeti [9], [13], [14], [17]. By fitting an existing movement model [9] to resource availability and wildebeest distribution data, Holdo *et al.* [14] found support for the hypothesis that wildebeest track the seasonal availability of intermediate forage biomass in the Serengeti (by maximizing green grass intake), but also identified an underlying fixed gradient of plant N content as an additional driver of the migration. The combination of intermediate biomass and high protein content of grasses in the Serengeti plains make them an important resource during the wet season. The remnants of green biomass in the northern woodlands provide a nutritional refuge during the dry season; by being able to track and exploit high-quality forage throughout the annual cycle, therefore, migratory wildebeest can substantially increase their nutritional input and reproductive output compared to sedentary animals with similar metabolic and nutritional demands. Disrupting the adaptive migratory movements of wildebeest can be expected to reduce effective carrying capacity, and reduce the ability of the ecosystem to sustain large numbers of ungulates. This was recognized by Owen-Smith [17], who used a mean-field model of herbivore population dynamics to predict that fragmenting the Serengeti landscape would have a dire impact on the size of the wildebeest population that the system could support.

**Figure 1:**
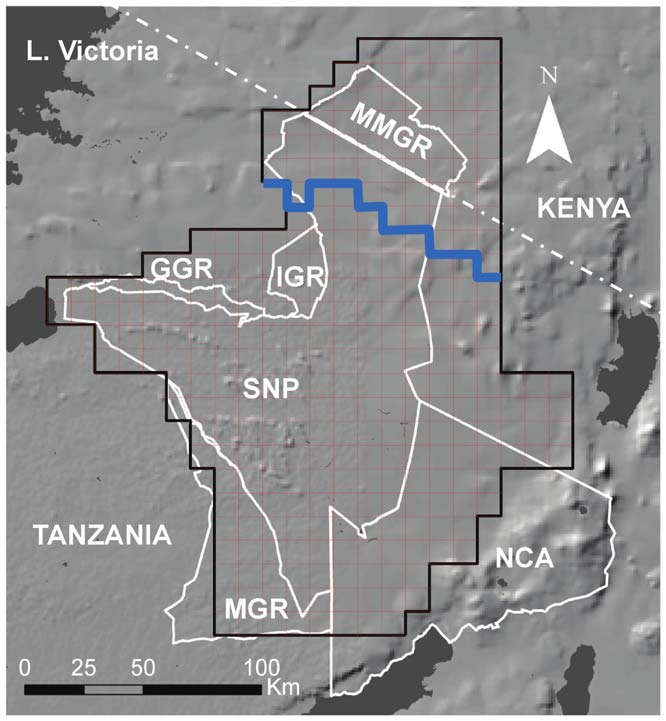
Map of the Serengeti ecosystem showing protected areas and geographic features. The SD model lattice is shown as a red grid (with the extent of the simulated ecosystem in black), with the modeled approximation to the proposed road in blue. The road divides the ecosystem into northern and southern components. Key to abbreviations: SNP  =  Serengeti National Park, NCA  =  Ngorongoro Conservation Area, MMGR  =  Masai Mara Game Reserve, MGR  =  Maswa GR, IGR  =  Ikorongo GR, GGR  =  Grumeti Game Reserve. Water bodies are shown in dark grey, and topography in lighter shades of grey.

Here, we use the specific road proposal that has recently been laid out for the Serengeti to explore the potential impact of developing barriers to migration (Fig. 1). We draw on a geographically-realistic model of the system, in which fragmentation of the habitat into two pieces is assumed to occur, precluding migration across the barrier provided by the road, but no actual habitat loss. Also, to better understand the role of movement in driving population size, we contrast our findings with a null model of no migration, in which we force the wildebeest to become sedentary and confined to restricted home ranges, with no movement even within the two pieces separated by the road. Although this does not represent a real scenario (e.g., wildebeest are unlikely to try to persist in the plains in the absence of surface water during the dry season), it serves to illustrate the potential role of movement in determining ungulate carrying capacity.

## Results

**Figure 2:**
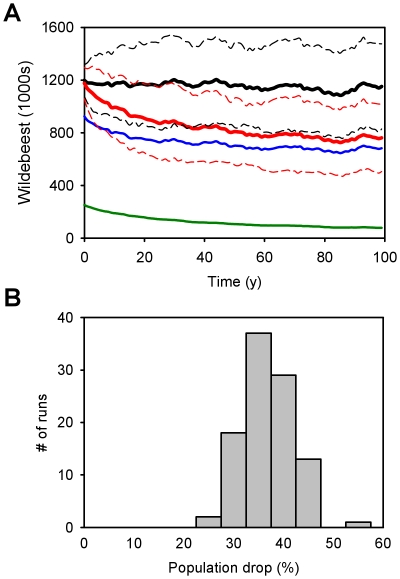
Simulated long-term effects of a barrier to migration across the northern Serengeti. **A**) Mean (100 runs) population sizes for the no barrier (black) and barrier scenarios (red), with the northern (green) and southern (blue) subpopulations of the barrier scenario included for reference. The standard deviations for the barrier and no barrier scenarios are indicated with dashed lines. **B**) Distribution of values for population decline for the barrier scenario across 100 simulations. Identical rainfall regimes are assumed for the barrier and no barrier scenarios in any given run.

**Figure 3:**
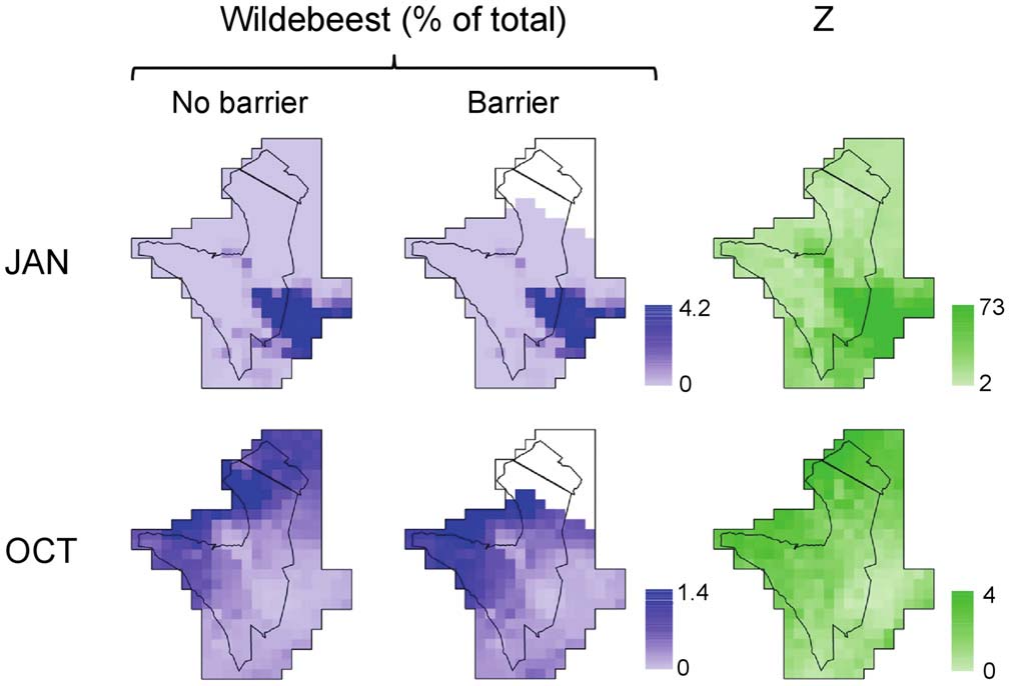
Simulated seasonal distributions of wildebeest and resources across the landscape. The wildebeest panels show the percentage of the total population that occupies each cell in the lattice (based on month-end counts) in the wet (January) and at the end of the dry (October) seasons for the no barrier and barrier scenarios. The resource panels show the mean daily values of Z (Eq. 1 in the text) across the landscape for the no barrier scenario.

**Figure 4:**
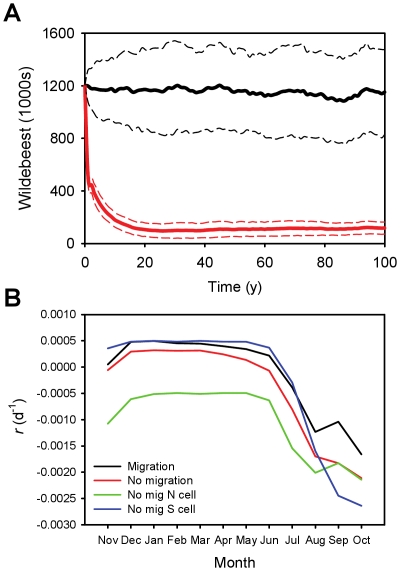
Simulated effects of movement on wildebeest population size in the Serengeti: **A**) mean (100 runs) population size for the default (no barrier, migration/movement allowed) scenario (black) from Fig. 2 and a no migration scenario in which wildebeest are treated as residents and prevented from moving among lattice cells (red). The standard deviations for each scenario are indicated with dashed lines. **B**) Mean monthly per capita population change (*r*) weighted spatially by wildebeest occupancy during the initial 10-year period of population collapse shown in **A**: the migration and no migration scenarios are contrasted, as well as values of *r* for individual cells from the northern woodlands (N cell) and southern plains (S cell) from the no migration scenario.

Model simulations predicted that the imposition of a barrier to migration at the site of the proposed road construction could plausibly cause significant drops in the wildebeest population. The simulated barrier caused a mean drop in population size of 35% (SD = 5%, N = 100 runs, Fig. 2) compared to having no barrier. Even though the highly-stochastic nature of rainfall in the system imposes a great deal of uncertainty in model outcome (Fig. 2A), the comparison of the barrier and no barrier scenarios for a given set of rainfall conditions showed clear effects on population decline (Fig. 2B). Hence, even without any habitat loss or increase in poaching, a partial disruption of the wildebeest migration is predicted to negatively affect wildebeest numbers, as well a cause a shift in habitat use patterns during the dry season (Fig. 3). We contrast this with the results of the no migration scenario, which predicted a collapse of the wildebeest population from an initial (present-day) population of 1.2 million to less than 10% of that number over time (Fig. 4A). An examination of sites in northern and southern Serengeti helps identify the mechanism that links movement and population dynamics (Fig. 4B). During the population crash that results from restricting movement (the first 10 years of the simulation), resource availability *Z* is maximized during the wet season (Dec-May) in the South, and per capita population growth is much higher than for sedentary wildebeest with home ranges restricted to the North, but the opposite is true late in the dry season (Sep-Oct). When averaged across the entire population (the red line in Fig. 4B), the sedentary strategy is outperformed by the migratory one, and the latter sustains a substantially greater population. Per capita population change eventually equalizes in the two scenarios, but at far lower population density in the no migration case.

The global sensitivity and uncertainty analysis (Table 1) suggested that our conclusion that the wildebeest population will decline as a result of barrier construction is robust (Table 2). The 95% confidence interval (across 1000 iterations) for population size, even without a barrier present, ranged from complete extinction to over 2.5 million animals (Table 2). Still, when controlling for rainfall regime, the no barrier scenario consistently outperformed the barrier scenario (Table 2), with a median population drop of 37% (95% CI: 16–73%). An examination of individual parameter effects suggested a contrast between effects on total population size and on predicted population drop following barrier construction. Wildebeest population size was most sensitive to: (1) uncertainty in grass production (rainfall effect on maximum grass growth ψ, a parameter that shifts the grass incremental growth curve towards the origin [thereby mimicking observed overcompensation effects] σ; (2) the decay rate of green grass δ_G_; (3) the slope for the effect of dry grass *D* on fire κ_1_) and quality parameters (q the power function that enhances the attractiveness of protein-rich grasses in Eq. 1); and (4) demographic parameters (*b_W_*) (Table 1). On the other hand, the three most important parameters for population decline caused by barrier construction were again ψ and σ (maximum grass growth and decay rate), but also ϕ, which is the parameter that influences the strength of the switching response that determines movement rate in Eq. 2. *I.e*., as ϕ increases, small changes in resource availability result in more movement across the landscape. This highlights the importance of the relationship between habitat fragmentation and mobility in determining population viability.

**Table 1:** Influence of parameter uncertainty on the size of the wildebeest population in the absence of a barrier (W) and the wildebeest response to the introduction of a barrier (ΔW).

				W†		ΔW†	
Parameter‡	Default value	SD or range	Error distribution	Adj. R^2^	S	Adj. R^2^	S
θ	1.39	0.0695	Gaussian	1.8	17.7	0.6	9.9
ψ	0.0167	0.002672	Gaussian	9.4	39.1	10.7	37.9
μ_0_	141	16	Gaussian	0.0	3.7	0.1	5.0
μ_1_	0.264	0.026	Gaussian	0.1	0.2	0.1	0.1
σ	46	14	Gaussian	22.5	60.2	23.0	55.4
ρ	0.5	0.15	Gaussian	0.1	5.7	0.2	6.8
δ*_G_*	0.061	0.006	Gaussian	6.5	32.7	2.0	16.9
δ*_D_*	0.0012	0.00012	Gaussian	0.1	1.8	0.0	3.5
*f*	0.42	0.042	Gaussian	0.9	12.4	0.3	7.3
κ_1_	0.061	0.04	Gaussian	4.7	27.8	2.6	19.0
κ_2_	3.72	2.4	Gaussian	1.8	17.7	1.5	14.8
α*_W_*	10.5	1.05	Gaussian	0.3	8.3	0.0	4.4
β*_W_*	9.9	5–10	Uniform	0.9	13.0	0.1	5.2
*dvi_G_*	5.4	0.54	Gaussian	0.0	3.1	0.0	3.5
*dvi_D_*	4.4	0.44	Gaussian	0.1	0.3	0.1	2.1
*b_W_*	0.24	0.026	Gaussian	7.8	35.7	2.6	18.8
*m_W_*	0.00049	0.000015	Gaussian	1.3	15.2	0.7	10.4
*a_W_*	0.0032	0.000096	Gaussian	1.5	16.2	0.6	9.8
*q*	3.15	0.16	Gaussian	4.7	27.6	1.3	13.7
ϕ	2	1–3	Uniform	0.4	9.2	3.1	20.6

Note: the results are based on 1000 iterations of the model run for 100 years for each parameter combination.

† Effects on W and ΔW are computed at the end of the run.

‡ Parameters are described in [28].

**Table 2:** Distribution of values for the size of the wildebeest population with and without a barrier generated by the global sensitivity analysis.

			Population drop	
Percentile	No barrier	Barrier	Absolute	%
97.5	0	0	57,800	16.2
95	41,600	19,400	91,400	18.9
75	519,500	316,300	215,800	30.4
50	908,200	580,400	311,900	37.1
25	1,355,800	918,700	444,800	43.6
5	2,311,200	1,670,600	714,800	62.1
2.5	2,629,300	1,971,600	825,700	73.1

Note: the results are based on 1000 iterations of the model run for 100 years for each parameter combination, assuming identical rainfall scenarios. Values have been rounded to the nearest 100.

## Discussion

One key factor underlying the superabundance of migratory ungulate populations in Serengeti (and elsewhere) is the ability of animals to efficiently track spatiotemporal variation in resource availability across landscapes with strong but noisy resource gradients [11], [14], [16]. In Serengeti, a barrier to migration would disrupt the natural seasonal patterns of habitat use across these gradients. The short-grass plains in the South of Serengeti have a short growing season and are unsuitable for year-round occupancy, but do provide a vital source of low-fibre/high-protein grass during the wet season. This is captured by the simulated maps of Z in the shown in Fig. 3. Conversely, the relatively dystrophic northern habitats of the Serengeti produce abundant low-quality biomass; while these areas are less nutritious during the wet season, they provide a refuge of last resort (Fig. 3) during times of dietary stress at the end of the dry season [18], and effectively act as key resource areas [19] for wildebeest and other migratory ungulates. The fragmentation of the landscape that is likely to result from road construction has the potential to gradually decouple the productive grasslands of the Serengeti plains from this dry-season refuge over time, and in the worst case, to dissect the system into separate habitats. In either event, the consequences of fragmentation are a loss of functional heterogeneity and a lowering of the carrying capacity of the system [15]. Similar conclusions have been reached about the ability of fragmenting African rangelands to sustain livestock numbers [20].

It should be acknowledged that a road might not by itself present an insurmountable barrier to migration, and therefore our model presents one possible scenario as far as the effects of the road on movement are concerned. There are reasons to believe, however, that as road traffic increases, fences and development might follow, eventually rendering a simple road project into a *de facto* barrier [6]. Our model suggests that such a barrier would render the wildebeest population markedly more vulnerable to significant declines in its numbers, even without drought, and that such effects are magnified by droughts—which are inevitable in this system over any reasonable time horizon.

Like all predictive models, the tool we present here inevitably has limitations. For example, even though we allow for environmental stochasticity (which introduces a substantial amount of uncertainty into our model output) in our simulations, we lack precise estimates of process error (mainly demographic stochasticity). We also still lack a specific mechanistic understanding of the importance of high-quality resources for birth rates in the plains during the wet season, and have had to infer the link between resource availability and population change through model fitting, as we have (for example) the effect of trees on grass biomass [14]. Our model, like others [21], assumes that births are constant and that only mortality is resource-dependent. This is because higher-quality data are available to correlate dry-season mortality with rainfall than to infer the mechanistic basis of variation in birth rates [22]. The Serengeti wildebeest have a well-defined birthing season lasting a few weeks during the wet season [23], and though markedly less variable than deaths, births have been observed to decline over time as the population has increased [22], suggesting density-dependent regulation of birth rates. We assumed that only mortality is variable and resource-dependent, and also assumed that births occur year-round. The second assumption alters the shape of the seasonal per capita population growth curves in Fig. 4B somewhat, but the cumulative differences between the migratory and non-migratory strategies are still valid. Still, it is clear that identifying more clearly the role of grass biomass and quality on pregnancy and birth rates is critical for deriving more refined predictions of future population trajectories. For the time being, we must rely on correlations between food biomass/quality and net population growth to model population dynamics. It is unlikely that such refinements, however, would markedly alter our qualitative conclusions.

These and other caveats do add uncertainty to our predictions. As a counter-argument, the model assumes that wildebeest would instantaneously adjust to a more restricted landscape and seek to maximize resource acquisition without attempting to cross the road. It predicts that as wildebeest in the South become deprived of the northern Serengeti and Mara habitats following barrier construction, they would automatically compensate for this loss by using more of the Western corridor, rather than aggregating at the now-truncated northern boundary of their altered range (Fig. 3), with potentially disastrous consequences. It remains unclear how plastic the migratory behavior really is and to what extent the wildebeest may be actually able to adjust to dramatically new conditions (*i.e*., to what extent does the migration obey relatively fixed cues shaped over evolutionary versus ecological time scales?). Strong hard-wired components in behavior may govern important aspects of long-distance migratory movement, with local cues driving movement within the plains and woodlands, for example [14]. If this is the case, the simulation results would drastically underestimate the impact of cleaving the spatial integrity of the Serengeti into these two habitats by the proposed road. We have also ignored the potential deleterious effects of other aspects of road construction, such as greater access for poachers [6], an important consideration given the fact that the size of the Serengeti protected area substantially buffers it from poaching impacts at present [24], [25]. These are all areas for further research and model improvement. In the meantime, the present model provides a rare quantitative tool for investigating the likely impact of disrupting the migration on the Serengeti wildebeest population.

Given the iconic importance of the wildebeest migration, both for its tourism potential and ecological significance, we advocate further research on the potential consequences of habitat fragmentation. Other models, both simpler [17] and more complex than ours (such as the SAVANNA model [26], [27]) have been or could potentially be applied to this problem, and an ensemble modeling approach would potentially provide a more robust evaluation of the range of risks associated with road construction. For example, despite our overall prediction of population decline with barrier construction, our results are more conservative than were previous estimates generated by the simpler mean field model developed by Owen-Smith [17]. Part of the reason for this might be the ability of the southern subpopulation in our geographically-realistic model to access reasonably wet portions of the ecosystem south of the road during the dry season, as opposed to projections based on the simpler two-compartment (Mara versus plains) implementation of the earlier model [17]. Additional approaches could resolve these discrepancies – but it should be noted that all of our results suggest that the expected fragmentation resulting from road construction would not have strongly negative consequences for the keystone wildebeest population and thus much of the rest of the Serengeti ecosystem.

## Materials and Methods

### Study system and modeling framework

The Serengeti ecosystem extends over more than 30,000 km^2^ in Tanzania and Kenya, with the Serengeti National Park as its dominant feature (Fig. 1). Here we define the ecosystem as the polygon defined by the extent of the wildebeest migration (Fig. 1). The migration is driven by two abiotic gradients: a seasonal rainfall gradient that increases from the Serengeti plains in the southeastern portion of the ecosystem towards the northwestern woodlands near Lake Victoria, and an opposing gradient of increasing soil fertility. During the wet season, between December and April, the wildebeest seek high-protein grasses in the plains, but as the dry season progresses, they shift towards the wetter woodlands in search of remaining pockets of green (but low-quality) forage.

To investigate the effect of imposing movement constraints on wildebeest population dynamics, we used a recently-published model of savanna herbivore, vegetation, and fire dynamics, the SD model [14], [24], [28]. This is a discrete-time model that partitions the ecosystem into a spatially-realistic grid with a spatial resolution of 10 km, and tracks the dynamics of grass, wildebeest movement and population dynamics, fire, and tree dynamics in each lattice cell. Environmental stochasticity is introduced through the random generation of monthly rainfall surfaces. The surfaces were generated by interpolating rain gauge data from the historical record for the period 1960–2006. To preserve intra-annual spatiotemporal correlations in the data, 12-month runs spanning complete wet and dry season cycles were kept as a single unit. Model simulations draw these units or rainfall “years” (which actually extend from November to October) randomly from the record.

In the model, grass production and decay are functions of rainfall (both seasonal and monthly) and grazing intensity. Two components are tracked in each cell: green and dry grass. The protein content of the former is dictated by a separate layer of ecosystem-wide grass N content, developed from field data. Wildebeest move at a weekly time step across the landscape, and their movements and local population growth are determined by a quantity we call *Z*, an index of resource availability. Previously, we used a model selection approach to derive the form for *Z* that best fit observed wildebeest movement data. A function of green forage intake (*I_G_*) and green forage protein content (*N*) provided the best fit:
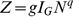
(1)


Here, *g* is the proportion of each cell occupied by grass (a function of tree cover, which for simplicity and to limit sources of uncertainty we keep constant in the present simulations) and q is a parameter. Wildebeest emigration from a lattice cell (Θ) is a function of local resource availability *Z* and expected *Z* across the entire landscape, E(*Z*):
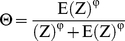
(2)


Emigrating wildebeest distribute themselves proportionately throughout the subset of target cells in the landscape with greater *Z* than the cell they have left. In our initial version of the model [28], movement and local population dynamics were slightly decoupled. The former was a function of Z and the latter of Z/W, as follows:
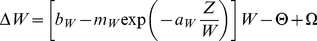
(3)


In eq. 3, ΔW is the change in wildebeest population density in a given lattice cell at each time step. This is a combination of local population dynamics (the first term on the r.h.s.) minus emigration Θ plus immigration Ω from neighboring cells. The implementation in eq. 3 independently provides good fits to movement data and population dynamic data, but it is largely phenomenological in that the factor that drives movement (Z) differs from the factor that maximizes per capita population growth (a proxy for fitness). A side effect of this is that simulated wildebeest do not necessarily make movement “choices” that maximize fitness. To make the model more mechanistic and internally consistent, we replaced Z/W on the r.h.s. of eq. 3 with Z:
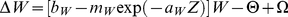
(4)


This required a recalibration of parameter *a_w_* from 0.21 to 0.24 in eq. 3 to produce a long-term wildebeest population of 1.2 million under “normal” conditions. This is the mean steady-state size of the Serengeti wildebeest population post-rinderpest (when disease kept the population in check). We kept all other model parameters unaltered with respect to earlier model versions. The full set of model equations and parameters is given in [28].

### Model scenarios

We used the SD model to make long-term (100-year simulations) projections of wildebeest abundance for both “no barrier” (the status quo), and “barrier” scenarios, under which the proposed road acts as a physical barrier to migration and cleaves the ecosystem into two separate habitats: a Northern compartment comprising 6,700 km^2^, and a Southern compartment comprising 24,000 km^2^, or 22 and 78% of the current extent of the migration, respectively. Both of these compartments contain mixtures of open grasslands (mainly in the southern plains) and woodland with variable amounts of tree cover. To simulate the presence of a barrier, we split the model lattice into a northern and southern compartment, with the size and shape of the compartments determined by the proposed road layout [6] (Fig. 1). When no barrier is present, wildebeest are able to move freely across the entire landscape according to eq. 1, but when a barrier is present, we assumed that the southern and northern subpopulations only move within their compartments. To test for an effect of the barrier on wildebeest population size, we conducted 100 model runs, each with randomly-drawn rainfall time series (but with identical time series applied to the barrier and no barrier scenarios for each run), and calculated the percent deviation in final wildebeest population size between the two scenarios (for the barrier scenario, the sum of the northern and southern sub-populations).

We also simulated an extreme “no migration” scenario that effectively prevents the wildebeest from moving among lattice cells, essentially forcing them to become sedentary, *i.e*., groups of wildebeest can forage within their 100 km^2^ home ranges (lattice cells), but not in adjacent cells. Though not necessarily a realistic situation, the no migration scenario serves both as a useful theoretical experiment (a null model of sorts) for isolating the role of movement in regulating carrying capacity (by completely eliminating movement), and for understanding quantitatively what a “worst-case” fragmentation scenario might look like in a migratory system were movement to be severely restricted (e.g., through fence construction, land cover change, or further road construction). For both the default (migration with no barrier) and no migration scenarios, we conducted 100 runs for 100 years. To understand better the mechanistic basis of differences in population dynamics between the migration and no migration scenarios, we calculated the simulated per capita population change on a monthly basis, both across the entire lattice (weighted by the relative abundance of wildebeest in each cell) and in two lattice cells with high wildebeest abundance in the dry season (a northern cell) and in the wet season (a southern cell). This allowed us to compare the relative performance of the average resident and migratory wildebeest with resident wildebeest at the two extremes of the migratory range.

### Global sensitivity and uncertainty analysis

To examine uncertainty in model predictions as a function of uncertainty in the parameters, we conducted a global sensitivity and uncertainty analysis by drawing values for 20 model parameters from normal (Gaussian) or uniform distributions. In both cases, the means of the parameter distributions were centered on their default values (Table 1). Standard deviations and ranges for the distributions were based on the literature and on the sampling distributions of parameters fit to data during model construction [28]. Many of the SD model parameters were derived in a hierarchical fashion by fitting model components (e.g., grass production, wildebeest movement and population dynamics) to data. We refit these distributions with the original model using maximum likelihood. We obtained multivariate 95% confidence bounds for the maximum likelihood estimates (MLEs) of parameter sets (e.g., *b_W_*, *m_W_*, and *a_W_* from Eq. 3 fitted together to a time series of wildebeest population size, or κ_1_ and κ_2_ fitted simultaneously to historic fire data) from sampling distributions. We generated these by drawing parameter values from 1000 iterations (following convergence) of the Metropolis algorithm [14]. For a given cluster, parameter values that result in a log-likelihood 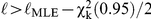 (where *k* is the number of parameters being estimated) are within the multivariate 95% confidence interval [29]. We then calculated standard deviations for the distributions (in the Gaussian case) and used these to sample parameter space in the sensitivity analysis (with zero truncation for nonnegative parameters). This approach is only an approximation and has potential drawbacks: for example, the correlation structure within sets of parameters (e.g., *b_W_* and *m_W_*) is ignored, as is the hierarchical nature of model construction and temporal autocorrelation in model fits to longitudinal data, and some “likelihoods” involve model fits to regression models obtained from the literature (e.g., a linear fit of grass production as a function of rainfall from [30]) rather than to raw data. These issues may under- or overestimate parameter uncertainty, but it was the best approach available given the assumptions built into our model (e.g., that mortality is a function of Z).

Once we had constructed parameter error distributions, we ran 1000 iterations of the model using random deviates from these distributions, with all 20 parameters being sampled in each iteration. We ran both the barrier and no barrier scenarios as before and calculated the absolute and relative drop in wildebeest numbers as a result of barrier construction at the end of 100 years. We conducted both simple regressions of the response variables against each parameter with R (v2.7.1) and used adjusted-R^2^ values and the slopes of the regressions to quantify the effect of uncertainty in each parameter. We derived a standard measure *S* of parameter influence:
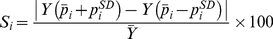
(5)where *S_i_* is the value of *S* for parameter *i*, 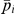 and 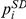 are the mean and standard deviation of parameter *i*, 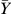 is the mean of the response variable (absolute or relative change in wildebeest population size), *Y*(*p_i_*) is the value of *Y* at parameter value *p_i_*, obtained from the regression equation [24].
